# Antioxidant in Food Safety and Sustainability

**DOI:** 10.3390/foods11030433

**Published:** 2022-02-01

**Authors:** Urszula Złotek, Anna Jakubczyk, Urszula Gawlik-Dziki

**Affiliations:** Department of Biochemistry and Food Chemistry, University of Life Sciences in Lublin, 20-704 Lublin, Poland; anna.jakubczyk@up.lublin.pl (A.J.); urszula.gawlik@up.lublin.pl (U.G.-D.)

Nowadays, safety and a positive effect on health are desirable features of food in addition to its nutritional value. Due to the growing awareness of consumers in this field, scientists and food producers are constantly looking for new solutions to increase the safety and bioactive properties of their products. Antioxidant properties that are assigned to groups of active compounds, i.e., phenolic compounds, carotenoids, essential oil components, bioactive peptides, or vitamins, are widely studied as health-promoting properties of food products. These compounds neutralize oxidative stress, which has an important role in the pathogenesis of cardiovascular diseases, cancers, neoplastic diseases, or Alzheimer’s disease. Antioxidants can also increase food safety by protecting food ingredients from oxidative and quality changes. The use of natural antioxidants especially is a new approach towards food technology [1,2,3]. Raw materials of plant origin, i.e., herbs, fruit, vegetables, etc., are particularly valuable sources of natural antioxidants [2,4,5,6].

It is well known that the content of bioactive compounds in plant raw materials depends on many factors related to the cultivation system, plant species, or the method of extracting biologically active compounds. The results obtained by Capistrán-Carabarin et al. [3] indicate that the content of phenolic compounds and the antioxidant properties significantly differed between species and accessions of *Phasoelum coccineus* and *Phasoelum vulgaris*. They also differed between various parts of the studied plants, i.e., higher concentrations were found in the seed coat than in the cotyledons in both species. Many factors have been indicated as important for extraction of phenolic compounds, i.e., the type of solvent, temperature, pH, or extraction time. However, the degree of water mineralization as a factor in the water extraction of phenolic compounds process seems to be poorly studied [4]. The research described by Wyrostek and Kowalski [4] shows that, in the case of herbal and tea extracts, the highest efficiency in the extraction of phenolic compounds with the use of water as a solvent is obtained with the use of deionized water and waters with medium levels of mineralization. Additionally, brews prepared with these types of water in the majority of cases possess the highest antioxidant activity [4].

The fortification of food products with components that possess antioxidant properties may also be used to enhance their pro-health properties. The supplementation of some food products with natural antioxidants is a new approach towards food technology connected with the production of functional food. Various groups of raw materials rich in bioactive compounds—for example, shrimp by-products [1], herbs [2], fruit and fruit juices [5], and probiotic bacteria [7]—can be used for food enrichment. The concentrated astaxanthin lipid preparation recovered from shrimp shell by-products added in an amount of 1% to cheese significantly increased its antioxidant properties and caused lower levels of lipid oxidation products during 4 weeks of storage [1]. 

Supplementation of flour products, i.e., cookies [2] or wafers [5,6], with plant raw materials rich in phenolic compounds resulted in an increase in antioxidant and other bioactive properties, i.e., potentially anti-inflammatory, antihypertensive, or antimicrobial activity. In the cited studies, the potential bioavailability of bioactive compounds and health-promoting properties of fortified products was confirmed by testing the potential bioavailability in the conditions of a simulated gastrointestinal tract.

As shown by in vivo studies, a diet rich in compounds with antioxidant activity contributed to immune modulation in personalized therapy for metabolic syndrome, influencing positively oxidative parameters and inflammatory condition biomarkers as well as biochemical parameters, which correlated with the reduction in the BMI value and the amount of visceral adipose tissue [8].

To conclude, compounds with antioxidant properties represent various chemical groups and have different origins. They may be a natural part of food or can be added into products in order to increase their health-promoting potential. Moreover, they play a very important role in human health and the maintenance of the quality and safety of food products; therefore, they should be the main component of the diet and functional food.
